# Vancomycin-Loaded Nanoparticles Enhance Sporicidal and Antibacterial Efficacy for *Clostridium difficile* Infection

**DOI:** 10.3389/fmicb.2019.01141

**Published:** 2019-05-24

**Authors:** Yi-Hsuan Chen, Tsung-Ju Li, Bo-Yang Tsai, Liang-Kuei Chen, Yi-Hsin Lai, Meng-Jia Li, Cheng-Yang Tsai, Pei-Jane Tsai, Dar-Bin Shieh

**Affiliations:** ^1^Department of Medicine, College of Medicine, National Cheng Kung University, Tainan, Taiwan; ^2^Institute of Oral Medicine and Department of Stomatology, National Cheng Kung University Hospital, College of Medicine, National Cheng Kung University, Tainan, Taiwan; ^3^Institute of Basic Medical Sciences, College of Medicine, National Cheng Kung University, Tainan, Taiwan; ^4^Department of Medical Laboratory Science and Biotechnology, National Cheng Kung University, Tainan, Taiwan; ^5^Center of Infectious Disease and Signaling Research, National Cheng Kung University, Tainan, Taiwan; ^6^Advanced Optoelectronic Technology Center, National Cheng Kung University, Tainan, Taiwan; ^7^Center for Micro/Nano Science and Technology, National Cheng Kung University, Tainan, Taiwan

**Keywords:** *Clostridium difficile*, spore, nanoparticle, target therapeutics, antibiotics

## Abstract

Current antibiotic treatments fail to eliminate the *Clostridium difficile* (*C. difficile*) spores and induce dysbiosis and intestinal inflammation via off-target effect, which causes refractory *C. difficile* infection raise an unmet need for a spore-specific antimicrobial treatment. We developed a sporicidal and antimicrobial vancomycin-loaded spore-targeting iron oxide nanoparticle (van-IONP) that selectively binds to *C. difficile* spores. Cryo-electron microscopy showed that vancomycin-loaded nanoparticles can target and completely cover spore surfaces. They not only successfully delayed the germination of the spores but also inhibited ∼50% of vegetative cell outgrowth after 48 h of incubation. The van-IONPs also inhibited the interaction of spores with HT-29 intestinal mucosal cells *in vitro*. In a murine model of C. difficile infection, the van-IONP significantly protected the mice from infected by *C. difficile* infection, reducing intestinal inflammation, and facilitated superior mucosal viability compared with equal doses of free vancomycin. This dual-function targeted delivery therapy showed advantages over traditional therapeutics in treating *C. difficile* infection.

## Introduction

*Clostridium difficile* (*C. difficile*) infection (CDI), the clinical manifestations of which (e.g., severe or bloody diarrhea, PMC, and toxic megacolon) are usually dangerous and fatal. *C. difficile* caused almost half a million infections in the United States in 2011, and an estimated 83,000 of the patients had at least one recurrence. Approximately 29,000 died within 30 days after the initial diagnosis (Bouwknegt et al., 2015). CDI is one of the most common healthcare-acquired infections associated with the rising use of antibiotics (Johnson et al., 1999). *C. difficile*, transmitted in humans through the fecal-oral route, is an anaerobic Gram-positive, spore-forming bacillus that produces toxins, including TcdA and TcdB, two large-molecules that damage intestinal mucosa and induce neutrophilic colitis and pseudomembranous colitis (PMC) (Leffler and Lamont, 2015). Moreover, more-virulent strains like BI/NAP1/027, an additional binary toxin composed of two subunits CdtA and CdtB, which increase the adherence of bacteria to the intestinal mucosa (Gerding et al., 2014).

The risk of *C. difficile* recurrence ranges from 20% after an initial episode to 60% after multiple recurrences. The healthcare costs associated with recurrent infection are usually much higher than are those from primary infections (Leffler and Lamont, 2015). Recurrences are primarily attributed to the dysbiosis and intestinal inflammation caused by current antibiotics like vancomycin (Gómez et al., 2017). Spore formation is also important for transmitting CDI because spores can withstand harsh environments, including radiation, high temperature, extreme freezing, and chemical disinfectants (Lawley et al., 2010; Deakin et al., 2012). Spores can re-activate themselves to the vegetative state via germination when the environment becomes favorable. Therefore, inactivating spores, increasing excretion of spores from GI tract, and inhibiting spore germination are the current unmet needs in CDI prevention and therapy.

To effectively prevent the transmission and recurrence of CDI, inactivation of spores is critical. Many nanoparticle types – silver, zinc oxide, and magnesium oxide – can inhibit spore dormancy and reactivation (Gopinath et al., 2015; Wagner et al., 2016). However, current sporicidal mechanisms rely on the release of the metal ions of the nanoparticles, which might be absorbed by the intestine. Therefore, an ideal sporicidal nanomaterial should target a specific spore but not release potentially toxic metal ions.

Furthermore, using antibiotics to eliminate newly germinated vegetative cells can reduce the infection rate of CDI. One approach for increasing the antimicrobial efficacy of antibiotics without raising the overall dose is to increase the local targeting concentration, particularly by conjugating antibiotics with pathogen-targeting delivery systems like nanoparticles. The targeting therapy concept originally comes from and is widely used in anticancer therapy (Sugahara et al., 2010; Simón-Gracia et al., 2016). This concept was gradually shifted to the antimicrobial field. In the regime of infectious disease, nanoparticles are conjugated with pathogen-targeting peptides or they act as pathogen-targeting materials themselves. For example, a *Staphylococcus aureus*-targeting peptide was conjugated to a nanoparticle platform loaded with antibiotics to treat difficult-to-manage infections in a mouse model (Kwon et al., 2017). The conjugation increases the local concentration of the antibiotics and reduces the off-target side effects. More important, the nanoparticle delivery platform appears to accumulate in the target site for longer period and able to penetrate deeper into infected tissue, which might also increase its antibacterial effects (Hussain et al., 2018).

We previously reported (Lee et al., 2017) that single-crystal non-stoichiometric Fe_3-δ_O_4_ magnetite nanoparticles (IONP) was sporicidal and was uniquely spore-binding. However, those nanoparticles were unable to inhibit *C. difficile* vegetative cells. On the other hand, vancomycin alone was unable to eradicate *C. difficile* spores; thus, outcomes were often relapses of the disease. In this study, we developed vancomycin-loaded Fe_3-δ_O_4_ magnetite nanoparticles (van-IONPs) to target and inhibit both the vegetative cells and the spores of *C. difficile*. We then comprehensively evaluated these van-IONPs *in vitro* and in a mouse *in vivo* disease model. We also explored the mechanisms of these synergistic inhibitory functions.

## Materials and Methods

### Preparation of the Fe_3-δ_O_4_ Iron Oxide Nanoparticles

Fe_3-δ_O_4_ iron oxide nanoparticles (IONPs) were synthesized using thermal decomposition, as previously described (Huang et al., 2011). Briefly, 1.42 g of iron acetylacetonate was mixed with 0.57 mL of oleic acid and 20 mL of trioctylamine (all 3: Sigma-Aldrich, St. Louis, MO, United States). The solution was refluxed at 325°C in an argon environment for 30 min. The solution was then cooled to room temperature and washed with a toluene-ethanol solution. A magnet was used to concentrate and collect the NPs, which were then transferred to chloroform solutions (Merck, Whitehouse Station, NJ, United States) and mixed with 0.4 mg/mL of prostate specific membrane antigen (PSMA) (Sigma-Aldrich) for 6 h at 55°C. Finally, the PSMA-coated NPs were collected and washed 3 times with high purity water (MQ water, 18.2 MΩ cm, Milli-Q; Merck Ltd., Taipei, Taiwan).

### Surface Modification of Nanoparticles With Vancomycin

The IONPs (6 × 10^15^ particles) were dissolved in 0.1 mL of 0.1 M 2-ethanesulfonic acid (MES) buffer with 0.5 M sodium chloride. The solution was then charged with 2 mg of carbodiimide (EDC) and 2.5 mg of N-hydroxysuccinimide (NHS) (all: 2 Sigma-Aldrich, St. Louis, MO) and mixed for 30 min at 4°C to activate the functional group for conjugation. After the reaction with EDC/NHS, the IONPs were attracted by a magnet and washed 3 times with MES buffer to remove residual EDC/NHS. The activated IONPs were then conjugated with 0.5 mg of vancomycin in 0.1 mL of MES buffer and stirred for 3 h at room temperature. The resulting vancomycin-loaded IONPs (van-IONPs) were centrifuged at 15,500 × *g* and the supernatant was collected for the free vancomycin quantification. The pellets were washed 3 times with 0.1 mL of MES buffer and finally dispersed in 0.05 mL of MES (concentration: 500 μg/mL) and stored at 4°C.

### Confirmation of Vancomycin-Modified Nanoparticles by Fourier Transform Infrared Spectroscopy

Fourier transform infrared spectroscopy (FTIR) was used to confirm that the vancomycin had been successfully conjugated on the surfaces of IONPs. The data were collected using the synchrotron radiation (SR-FTIR) spectromicroscopy facility at the National Synchrotron Radiation Research Center (NSRRC) beamline 14A1 (BL14A1) in Taiwan. An FTIR spectrometer (Thermo Nicolet 6700; Thermo Fisher Scientific, Waltham, MA, United States) and continuum infrared microscope were used to record the data (resolution: 4 cm^-1^, step size: 15 μm, aperture size: 30 μm, and 128 scans).

### Quantification of Vancomycin Loaded on Nanoparticles

Microplate readers (SpectraMax M2/M2e; Molecular Devices, San Jose, CA, United States) were used to measure the fluorescence emission spectra of vancomycin hydrochloride (Sigma-Aldrich) solutions. To plot the standard curve of vancomycin concentration, a 96-well plate was loaded with solutions (0.1 mL at vancomycin concentrations of 0, 0.0625, 0.125, 0.25, and 0.5 mg/mL). Fluorescence was measured using a 280 nm excitation wavelength and a 380 nm emission wavelength. 0.1 mL supernatants collected from the conjugation step were moved to a 96-well plate to measure fluorescence. The final concentrations of the supernatants were compared with the initial concentrations to calculate the binding amount of vancomycin on the van-IONPs.

### Bacterial Culture and Spore Purification

*C. difficile* CCUG 37780 (tcdA-, tcdB-) and BAA-1805 (tcdA+, tcdB+) were purchased from the Culture Collections of the University of Göteborg (Göteborg, Sweden), and from American Type Culture Collection (Manassas, VA, United States), respectively. All strains were incubated in brain-heart infusion medium (BHIS; BD Difco, Franklin Lakes, NJ, United States), which supplemented with 0.5% yeast extract (BD Difco) and 0.1% L-cysteine (Amresco, Solon, OH, United States), at 37°C under anaerobic conditions. The spores were prepared with modifications and then purified as previously described (Sorg and Sonenshein, 2010). Briefly, 0.2 mL of *C. difficile* in BHIS medium was spread on 6-well dishes with BHIS, and then the dish was incubated at 37°C in an anaerobic chamber for 7 days. After the incubation, the 6-well dishes were washed with 200 μL of ice-cold water to separate the cells on the dishes; the cells were resuspended in 3 mL of ddH_2_O. To further separate the spores with the residual vegetative cells, 100 μL of the suspension was spread on top of the 1 mL 50% Nycodenz^®^ solution in an Eppendorf tube, and then centrifuged at 15,500 × *g* for 30 min to separate spores from vegetative cells. The pellets were washed and centrifuged 5 times with ice-cold ddH_2_O to remove the Nycodenz, and then stored at 4°C.

### Spore Germination Assay

The stock solutions containing the spores were incubated at 60°C for 30 min and then the supernatant had been removed, the BHIS was added to a final spore concentration (15,500 × g) of OD600 0.2. Two microliters of solution were dispensed into each Eppendorf tube, which was then centrifuged at 15,500 × g to remove the BHIS. The pellets were resuspended in 100 μL of ddH_2_O test medium for the following groups: (1) free vancomycin (18 mg/mL), (2) bare IONPs (500 μg/mL), (3) the van-IONPs (IONP: 500 μg/mL; Equivalent vancomycin on such concentration of IONPs: 18 mg/mL), (4) 3% bleach (Wako, Osaka, Japan), and (5) ddH_2_O alone (negative control). After 20 min of incubation, each tube was centrifuged at 15,500 × *g*, washed 3 times with ddH_2_O, and then resuspended in 180 μL of BHIS. A 90 μL solution from each test sample was then transferred to a 96-well plate that contained 10 μL of taurocholic acid (TA)(Sigma-Aldrich) to the final concentration 10 mM to induce spore germination. The germination process was analyzed for 12 min at a wavelength of 600 nm (an optical density of 600: OD600) determined at 1 min intervals using a spectrophotometer (Tecan Austria GmbH, Grödig, Austria) at room temperature. The Tn and T0 denote the OD values at time n and time 0. Untreated spores were used as negative controls. Spore germination induction and analyses were modified from previously described protocols (Sorg and Sonenshein, 2010).

### Spore Viability Analysis

After the germination assay, 100 μL of solution was removed from the 96-well plate, serially diluted, and spread evenly on the BHIS dishes. The plate-counts (CFU/mL) were done after 48 h of incubation in an anaerobic tank at 37°C.

### Nanoparticle-Spore Binding Efficiency Assay

*C. difficile* BAA-1805 spores at a concentration of OD600 2.0 were incubated with 100 μL van-IONPs (75, 150, 300, 500, 700 μg/mL) in ddH_2_O in tubes for 20 min. The spore samples treated with ddH_2_O were mock controls. The samples were then placed next to a magnet for 20 min to transfer all the supernatant to clean tubes. The OD600 of the supernatants were measured and compared with the control. The binding efficiency was calculated as followed.

$$\mathrm{Efficiency}=\left(1-\frac{\mathrm{OD}600\;\mathrm{of}\;\mathrm{supernatant}\;\mathrm{of}\;\mathrm{experiment}\;\mathrm{group}}{\mathrm{OD}600\;\mathrm{of}\;\mathrm{supernatant}\;\mathrm{of}\;\mathrm{mock}\;\mathrm{control}}\right)\times 100\%$$

Then, the magnet-concentrated fractions were washed 3 times with ddH_2_O to remove the residual supernatant and resuspended in 100 μL of ddH_2_O. The spores were lysed via heating the van-IONP pellet-spore complex in 95 Celsius degree for 15 min to extract the DNA from spores (Rose et al., 2011). A polymerase chain reaction (PCR) was used to measure 1 μL of magnetically-concentrated fractions and of supernatants for the presence of the *TPI* gene, a *C. difficile* housekeeping gene. Images of spores and of van-IONP pellets in the supernatant were captured using a transmission electron microscope (TEM) (JEM-1400; JEOL, Tokyo, Japan).

### Spore Adhesion Assay

To assess the ability of our van-IONPs to reduce the adherence of spores to the intestinal epithelial cells, we used *in vitro* human intestinal epithelial cells (HT29). We adapted and modified the method previously described to assay the adherence of spores to the intestinal epithelial cells (Mora-Uribe et al., 2016). In brief, HT29 cells were incubated in the 24-well plate (1.8 × 10^5^ cells/well). *C. difficile* BAA-1805 spores (1.8 × 10^6^; MOI 10) were treated with FM 4-64 fluorescent dye, coincubated with van-IONPs for 20 min, and then added to the plate. The spores were added to the wells to infect the HT29 cells. The spore-infected cells were incubated for 3 h for spore adherence. After they had been incubated, the spores unbound to cells were washed away 3 times with PBS, and the HT29 cells remaining on the plate were examined under a fluorescence microscope. The fluorescence intensity was measured by ImageJ and the relative fluorescence was calculated by setting intensity of spore alone group as one.

### *In vivo* Analysis of the Efficacy of Vancomycin-Conjugated Nanoparticles

Animal studies were approved by the Laboratory Animal Center of the National Cheng Kung University Medical College (IRB #: NCKU-IACUC-105-182) and performed according to the local guidelines. To evaluate the *in vivo* efficacy of the targeted therapy, an infected mouse model was used. The protocol was adapted and modified from previous report (Hung et al., 2015). Briefly, the mice were given an antibiotic cocktail (0.4 mg/mL of kanamycin, 0.035 mg/mL of gentamicin, and 0.057 mg/mL of colistin) in their drinking water for 48 h, and then they were given an oral 200 μL proton pump inhibitor (PPI) (2 mg/mL) every 12 h for 2 days before they were infected with *C. difficile* spores. All the mice were pre-gavaged with a 50 μL PPI (2 mg/mL) and then intraperitoneally (i.p.) injected with clindamycin (4 mg/kg). *C. difficile* BAA-1805 spores (2 × 10^6^ CFU) were then coincubated with 0.1 mL 500 μg/ml van-IONPs, 0.1 mL 500 μg/ml IONP, 0.1 mL 18 mg/ml vancomycin and 0.1 mL ddH_2_O as spore alone group for 20 min to enable attachment of van-IONPs to the spores. before the mice were fed with sample solution through oral route. The antibiotic cocktail water was replaced with normal water after the *C. difficile* infection symptoms (e.g., diarrhea, weight loss, hunched posture, and death) were monitored. All mice were anesthetized with isoflurane with oxygen and their colon tissue was harvested 72 h later. TRIzol reagent (Sigma-Aldrich) was used to extract colon tissue RNA. The levels of inflammatory gene expression were estimated using a real-time PCR assay (StepOnePlus; Applied Biosystems). Histopathology was used to evaluate the damage caused by CDI to the intestinal mucosa. The colon samples were fixed in 4% formaldehyde buffered with PBS and then embedded in paraffin. Deparaffinized 6 μm-thick sections were stained with hematoxylin and eosin (H&E) and Periodic acid-Schiff (PAS) stains. The results were captured using an optical microscopy.

### Statistics

All data were expressed as the mean ± standard deviations and statistical comparisons among the groups were analyzed by Student’s *t*-test. Multiple intergroup comparisons were assessed by one-way ANOVA, followed by post hoc Tukey’s test with GraphPad Prism version 6.0. Statistical significance was set at *P* < 0.05.

## Results

### *Clostridium difficile* Spore Germination Assay of Free Vancomycin

**FIGURE 1:**
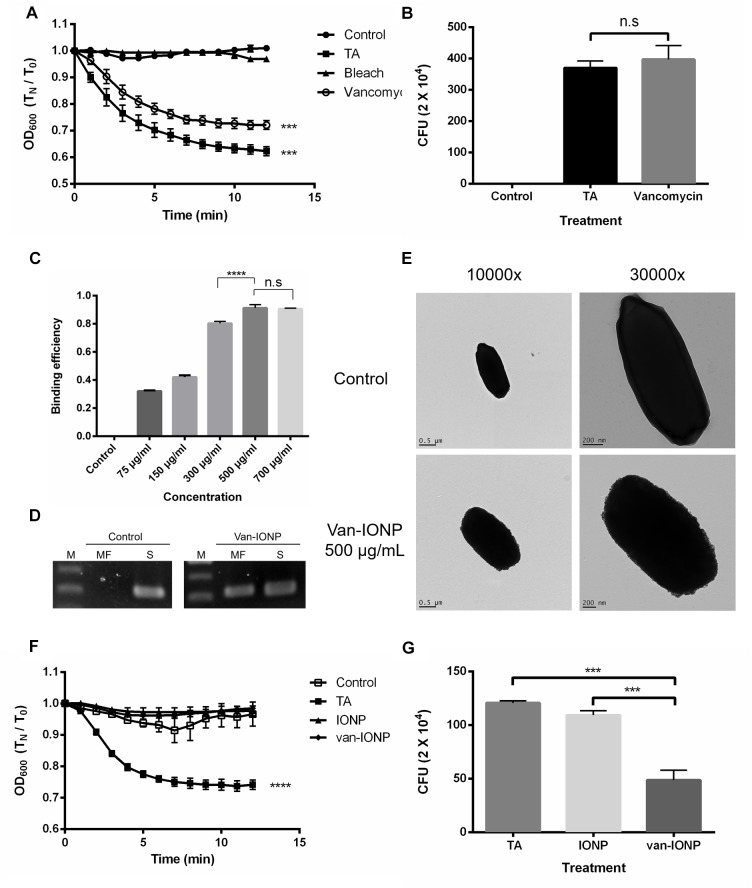
*In vitro* assay of the targeting property and therapeutic potential of van-IONPs. **(A)** The vancomycin treatment at 18 mg/mL did not significantly inhibit spore germination in the 12-min germination induction analysis. **(B)** There was no significant difference in the CFU count between the 18 mg/mL vancomycin-treated group and the 10 mM TA-treated group after 48 h of incubation. The result of both 12 min germination induction analysis and 48 h incubation showed that the free vancomycin was unable to specifically target the spores and to exert prolonged antibacterial effect on vegetative cell outgrowth. **(C)** The OD_600_ binding test shows the binding efficiency of van-IONPs to spores reach a plateau after 500 μg/mL. **(D)** The PCR assay of magnetically-concentrated fraction showed the presence of *TPI* gene, a housekeep gene of *C. difficile* spores, on the van-IONPs. (MF, magnetically-concentrated fraction; S, supernatant). **(E)** The upper TEM image shows that native spores have a smooth coat; the lower TEM images show accumulated van-IONPs on spore surfaces with rough surface after incubation and that the spores were completely covered with 500 μg/mL van-IONPs. **(F)** The van-IONP treatment can significantly inhibit the germination of spores in the 12 min germination induction analysis **(G)** The CFU of the vegetative cell outgrowth was counted by plating and incubating them on BHIS for 48 h and showed van-IONP is efficacious in inhibition the vegetative cell outgrowth. ^∗∗∗^*P* < 0.001, ^∗∗∗∗^*P* < 0.0001.

To show whether the free vancomycin can target spores and inhibit the spore germination as well as the following vegetative cells growth, an optical density-based spore germination assay was done. The CCUG 37780 spores after vancomycin treatment were washed 3 times with ddH_2_O and activated in 10 mM of taurocholic acid (TA) solution. Spore germination was not significantly inhibited after vancomycin treatment at 18 mg/mL concentration (Figure 1A). Colony-forming unit (CFU) assay showed no significant inhibition of spore germination 48 h after they had been treated with vancomycin at 18 mg/mL concentration (Figure 1B). The results suggest the vancomycin cannot specifically target to spores and to exert antibacterial effect (*n* = 4).

### Targeting the Efficacy of Vancomycin-Loaded Fe_3-δ_O_4_ Nanoparticles on *C. difficile* Spores

FTIR analysis (Supplementary Figure 1) and fluorescence spectroscopy confirmed the conjugation and loading capacity of the vancomycin and the IONPs (*n* = 5). Vancomycin has a fluorescence emission peak of 350 nm when excited by 280 nm photons (Zarkan et al., 2016). We obtained a standard curve and estimated that there were 5.8 ± 1.5 molecules of vancomycin on each IONP.

To estimate the dose dependence of van-IONP in spore binding, we perform the nanoparticle-spore binding efficiency assay (*n* = 4). The results showed that nanoparticles targeted to the spores were dose-dependent and reached a plateau at concentration of 500 μg/mL (Figure 1C). Furthermore, to confirm the spores were successfully captured by the van-IONP, PCR analysis was performed, and the result confirmed the presence of *C. difficile* spores on the surface of the van-IONPs. The band of the *TPI* gene, which is the housekeeping gene of *C. difficile*, showed that the van-IONPs captured the spores (Figure 1D). Based on the binding efficiency assay which showed a plateau after 500 μg/mL, we chose 500 μg/mL as our standard concentration of van-IONPs in the following experiments. The concentration of vancomycin on van-IONP was estimated to be 18 mg/mL.

The specific targeting effect of the van-IONPs on the *C. difficile* spores was also assayed using a transmission electron microscope (TEM). TEM images showed that spores treated with 500 μg/mL van-IONPs were fully surrounded by the IONPs (Figure 1E).

### *In vitro* Assay of the Therapeutic Potential of Vancomycin-Conjugated Nanoparticles

To investigate whether van-IONPs have inhibitory effect on spore germination, an optical density-based spore germination assay was done (*n* = 4). The spore germination was significantly inhibited by both van-IONP and IONP (Figure 1F). After germination, the spores were plated for the CFU assay. The CFU assay showed that after 48 h of incubation, the vancomycin on the van-IONPs had bound to the spores and that the growth of newly germinated vegetative cells had been inhibited (Figure 1G) The CFU assay showed that the vancomycin was brought to the surface of spores via van-IONP to inhibit the outgrowth of the vegetative cells.

**FIGURE 2:**
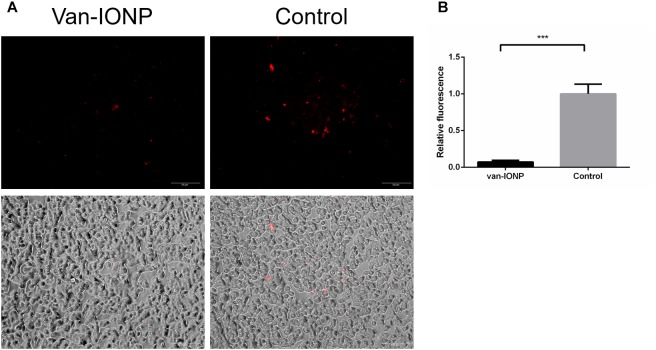
*In vitro* assay of intestinal protective potential of van-IONPs. **(A)** Fluorescence (upper) and light-overlaid (lower) micrographs of adhered fluorescent-labeled *C. difficile* spores (red) to monolayers of HT29 cells. The amount adhered to spores was lower in the van-IONP-treated group than in the control group. **(B)** The fluorescence intensity was counted using ImageJ software; the intensity was significantly higher in the control group than in the van-IONP-treated group (*n* = 5). ^∗∗∗^*P* < 0.001.

To further illustrate the protective effect of van-IONP to the intestinal mucosa, a spore adhesion assay (*n* = 4) was conducted and showed that spores treated with van-IONPs had fewer adherence with intestinal mucosal comparing with the control group (spore-only) (Figure 2A). The fluorescence intensity was measured by ImageJ and the control group was significantly higher than that of the van-IONP-treated group (Figure 2B). The result implied the van-IONPs can reduce the binding affinity of spores to the intestinal cells.

### *In vivo* Assay of Therapeutic Effects of Vancomycin-Conjugated Nanoparticles

**FIGURE 3:**
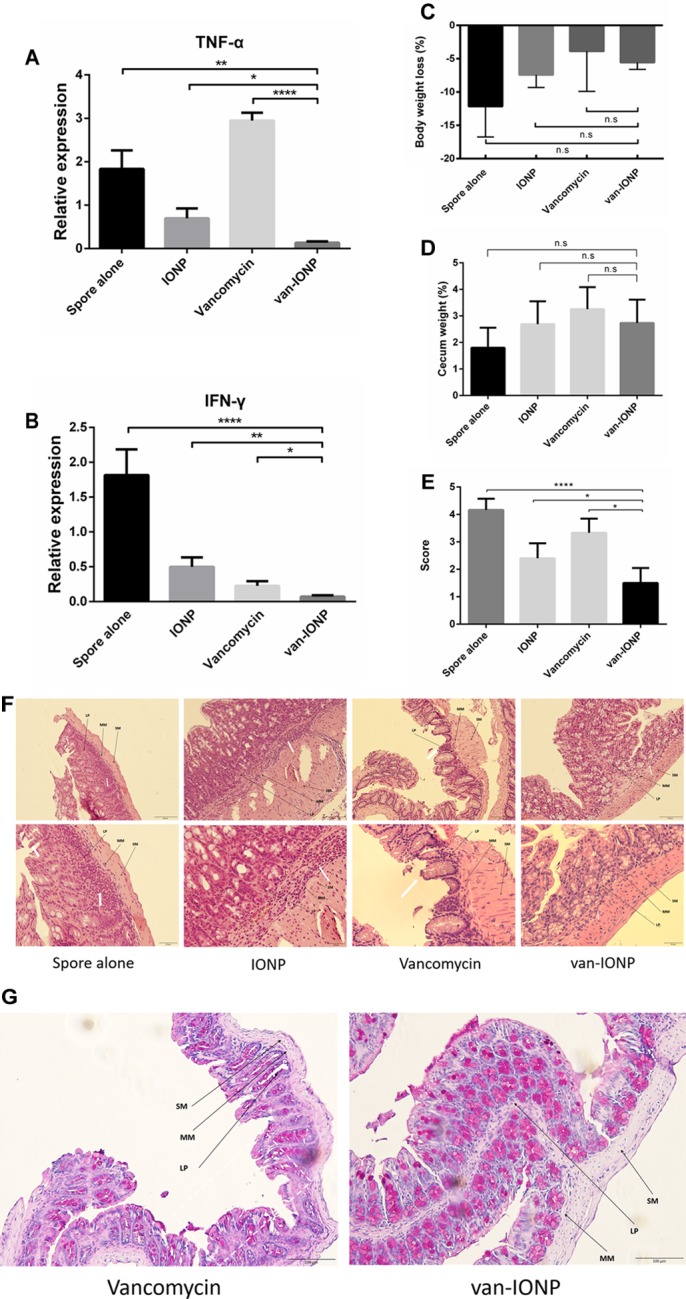
*In vivo* assay of therapeutic effects of van-IONPs in mice (*n* = 6). **(A)** The TNF-α expression level of the van-IONP was significantly lower than spore alone, free vancomycin and IONP group. TNF-α expression is highest in free vancomycin group which suggests the intestinal cell damage caused by free vancomycin. **(B)** The IFN-γ expression level was significantly lower than spore alone, free vancomycin and IONP group **(C)** The cecum weight of the mice was not significantly different between each group. **(D)** The body weight loss of the mice was not significantly different between each group **(E)** The pathological scores were measured by 6 randomly selected sections of intestinal tissue and the van-IONP group is significantly lower than other groups **(F)** Histopathological images showed lower neutrophil infiltration in the lamina propria (LP) and submucosa (SM) of van-IONP group compared to spore alone and IONP group. The mucosal layers of the free vancomycin group showed greater erosion than the van-IONP group. **(G)** PAS staining shows the decreased mucosal cell viability damaged by free vancomycin. (LP, lamina propria; MM, muscularis mucosae; SM, submucosa; white arrow, lesion site) ^∗^*P* < 0.05, ^∗∗^*P* < 0.01, ^∗∗∗∗^*P* < 0.0001.

To investigate the potential of the targeted therapy of van-IONPs in clinical translation, we compared the therapeutic effects of the free vancomycin, which is widely used to treat CDI, with vancomycin-conjugated nanoparticles. In our infection model (*n* = 6), the mice were orally infected by the toxigenic *C. difficile* BAA-1805 spores, resulting in 17% mortality after 3 days from infection without treatment. The inflammatory signal peaked 3 days after *C. difficile* infection had been induced (Lee et al., 2017). Thus, in our *in vivo* mice experiment, the mice were sacrificed 3 days after infection for analysis. A real-time reverse transcription quantitative polymerase chain reaction (RT-qPCR) was used to determine the gene expression of the proinflammatory cytokines TNF-α and IFN-γ. The inflammation level in van-IONP is significantly lower than spore alone, vancomycin, and INOP group. To note that, the expression level of TNF-α and IFN-γ in free vancomycin group is significantly higher than van-IONP group, suggesting that a high dose of free vancomycin can further increase inflammation level in CDI mice (Figure 3A,B). In the clinical outcome, both body weight loss and cecum weight have no difference between free vancomycin, IONP and van-IONP group (Figure 3C,D). The histopathological image showed lower neutrophil infiltration in van-IONP group compared to spore alone and IONP group (Figure 3F). The mucosal cells of free vancomycin showed more erosion than van-IONPs group (Figure 3F) and the PAS stain also showed more mucosal cell viability in mucus production in van-IONP comparing to free vancomycin group (Figure 3G). The pathological scores measured by the inflammatory cell infiltrate, epithelial changes and mucosal architectures in 6 random fields of tissue sections according to the suggested guideline (Erben et al., 2014). The result of van-IONP showed significantly lower score than other groups which indicate the therapeutic effects of van-IONP *in vivo* (Figure 3E).

## Discussion

*C. difficile* is a major cause of healthcare-acquired life-threatening diarrhea, which is associated with substantial mortality around the world (Ananthakrishnan, 2011). The traditional treatments of choice include the use of antibiotics metronidazole and vancomycin. However, the off-target effect of the traditional antibiotics can contribute to intestinal dysbiosis (aka: dysbacteriosis) and result in recurrent infection (Hopkins and Wilson, 2018). To reduce the off-target effect, one on the paradigms is to deliver the therapeutics via targeting vehicles. Antibiotics-loaded nanoparticles have been widely used in combating other strains of bacteria, such as e.g., *Staphylococcus aureus* (Alvarez et al., 2014; Kavruk et al., 2015; Hussain et al., 2018), and *Pseudomonas aeruginosa* (Kwon et al., 2017), to provide sustained and targeted delivery to enhance the increase therapeutic efficacy. However, the carrier nanoparticles of those delivery systems do not possess any therapeutic efficacy.

The interaction of *C. difficile* spores to the intestinal mucosa plays a significant role in many stages of the CDI infection: the initial stage of infection, the persistence of *C. difficile* spores in healthy individuals and the CDI relapse (Paredes-Sabja and Sarker, 2012). While vancomycin is highly active against vegetative cells, there is no evidence to show it has antispore activity (Baines et al., 2009); and although new antibiotics, fidaxomicin, was reported to inhibit sporulation, its inhibitory effect on preexisting spores is unclear (Babakhani et al., 2012).

Because *C. difficile* infection contains spore germination phase and vegetative outgrowth phase, we developed a dual-function targeted therapy using van-IONPs by conjugating IOPNs with vancomycin to inhibit germination as well as outgrowth. We firstly confirmed that IONPs have spore-specific binding properties that make them useful for the targeted delivery of synergistic therapeutics. TEM images showed that our van-IONPs had surrounded the spores even after they had been rigorously washed, which indicated the strong binding affinity. The mechanism by which the van-IONP can possess strong binding affinity to the spores is still not very well understood. It is probably due to the hydrophobic interaction between the exosporium and the nanoparticles (Joshi et al., 2012; Knowles et al., 2018). The van-IONP also showed inhibitory effect on the spore germination in the germination assay. One of the possible mechanisms is the interference of the CspC receptor pathway caused by surrounded nanoparticles that are needed in the spore germination, which is a bile salt sensitive receptor and plays an important role in initiating the spore germination (Francis et al., 2013).

Moreover, several studies report that immobilization of antibiotics on nanocarriers can be a general strategy for enhancing the concentration and potentiate the antimicrobial effect to the surface of pathogens (Hassan et al., 2017; Hussain et al., 2018). In our study, the van-IONPs and the conjugated vancomycin surround the spore very densely under the TEM images. The bioavailability of conjugation of vancomycin to the surface of nanocarriers through EDC/NHS reaction has been shown in past studies and the vancomycin can still preserved the functional groups to form specific hydrogen bonding toward gram-positive *C. difficile* vegetative cells at the terminal d-alanyl-d-alanine moieties (Gu et al., 2003; Kell et al., 2008). We found that the van-IONPs significantly inhibited the outgrowth of germinated spores after 48 h of incubation whereas the IONPs and free vancomycin cannot. The result showed vancomycin can be brought to the surface of spores by our spore-targeting van-IONPs. This local aggregation of van-IONP around spores may concentrate the vancomycin dosage around the spores (Qi et al., 2013; Hassan et al., 2017). We also observed that under the same concentration, the van-IONP can inhibit outgrowth of vegetative cells and IONP cannot (Supplementary Figure 2). Thus, our van-IONPs not only inhibit spore germination, but also enable vancomycin to accumulate on the spore surface, which optimizes its antibacterial efficacy to outgrowth of vegetative cells. This dual-function design is necessary to solve the unmet needs in current CDI therapy, which cannot deal with the spores.

Studies showed the outer layer of the *C. difficile* spores contain enterocytic-surface-specific ligands and receptor that provide specific adherence to the intestinal mucosal cells. The hydrophobicity of the intact exosporium of the spores also contribute to stronger attachments to the cells. The alternation to the ultrastructure of the exosporium reduced the hydrophobicity and adherence (Joshi et al., 2012; Paredes-Sabja and Sarker, 2012; Mora-Uribe et al., 2016). We hypothesized the coating of the van-IONPs around the spores can interfere with the ligand-receptor and hydrophobic interactions between the exosporium and the intestinal mucosal cells and significantly reduce the number of residual spores in the gut. In our study, after three rounds of washing with buffer, lower amount of residual fluorescence of dyed spores was observed in the HT29 cells in van-IONP treated group. We found that vancomycin does not affect the IONP adherence to spore and can significantly reduce spore adherence to mucosal cells, which indicate the vancomycin is not important for reducing spore adherence (Supplementary Figure 3). In the present study, van-IONP can significantly reduce adherence of spores to the HT29 and show potentially protective effect to the intestinal mucosa.

The 22 nm IONP has been shown to have high biocompatibility in previous report (Lee et al., 2017) and conjugation of vancomycin through EDC/NHS reaction to nanoparticle won’t increase its toxicity (Qi et al., 2013). The vancomycin on van-IONP was also stable in the acidic environment of the mouse digestive tract. To evaluate the therapeutic effect and the benefit of specific targeting provided by our van-IONP, we analyzed the clinical severity and the inflammation levels in the mouse intestines. 500 μg/mL van-IONPs can significantly reduce severity of infection in inflammatory characteristic and pathological outcome comparing to other groups. However, there were no significant differences in total body weight loss or cecum weight between the groups. This might be associated with many confounding factors such as the uncontrollable intake of food and water of each mouse. The expression of interferon gamma (IFN-γ) and tumor necrosis factor alpha (TNF-α) is significantly lower in van-IONP group than in other groups, which showed the potential for van-IONPs as an effective therapeutic agent. The surge of necrosis factor alpha (TNF-α), which mediates the necrotic cell death pathway of the most cells (Morgan et al., 2008), in free vancomycin group suggested the excessive concentration of antibiotics might create reactive oxidative stress to the cells and induce mitochondrial dysfunction to initiate cell death (Kalghatgi et al., 2013). This is consistent with reports (Sekirov et al., 2008) that vancomycin might induce tissue damage, disrupt intestinal microbiota, and result in a more severe inflammation. Though vancomycin was effective in suppressing acute infection but the perturbation of antibiotics to the intestinal tissues lead to more susceptible to infection (Warren et al., 2013). The histopathologic analysis of the colon tissue showed more mucosal cell shedding in H&E stain in free vancomycin group and PAS staining also confirmed that mucus secretion was lower in the free vancomycin-treated CDI mice than in the van-IONPs-treated group, which indicated profound damage to the intestinal mucosa.

## Conclusion

**FIGURE 4:**
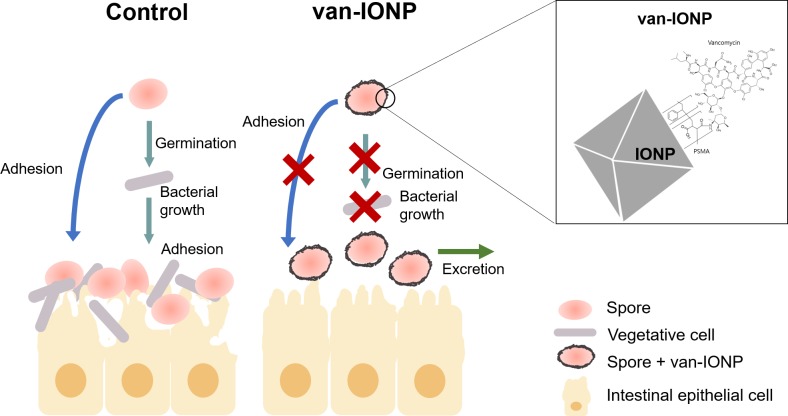
Schematic illustration of the potential mechanisms that underlay the dual-function efficacy of van-IONPs to *C. difficile* infection. Van-IONPs bound to spores and inhibited their germination and adhesion to intestinal mucosa, which increased clearance of the spores from the mucosa surface. The bounded van-IONP can also eliminate the newly germinated vegetative cells. This was enabled by conjugating vancomycin to the surface of IONPs (Box: top right) and creating a locally concentrated dosage of vancomycin around spores.

In conclusion, we developed a vancomycin-targeted delivery system against CDI in which the nanocarrier also inhibited spore germination. The underlying mechanisms that our van-IONPs utilized were shown in Figure 4 and include: (1) the van-IONPs strongly bind to *Clostridium difficile* spores and reduce their adherence to the intestinal mucosa; (2) biocompatible van-IONPs with a high loading capacity aggregates vancomycin around the pathogens; (3) the van-IONPs also inhibited spore germination. Finally, the van-IONPs could be scaled up using green synthesis processing at a significantly lower cost than current antimicrobial targeting biologics. Furthermore, the intriguing mechanisms underlying the specific interaction between van-IONPs and *Clostridium difficile* spores warrant additional investigation, which might lead to new prophylactic or therapeutic to a wide spectrum of other spore-generating pathogens in human and livestock diseases.

## Ethics Statement

This study was carried out in accordance with the recommendations of Laboratory Animal Center of the National Cheng Kung University Medical College (IRB #: NCKU-IACUC-105-182). The protocol was approved by the Laboratory Animal Center of the National Cheng Kung University Medical College.

## Author Contributions

Y-HC, P-JT, and D-BS designed the experiments. Y-HC, L-KC, Y-HL, C-YT, and M-JL conducted the experiments. Y-HC, T-JL, B-YT, and L-KC analyzed the data. Y-HC, T-JL, and B-YT prepared the manuscript.

## Conflict of Interest Statement

The authors declare that the research was conducted in the absence of any commercial or financial relationships that could be construed as a potential conflict of interest.

## References

[B1] Alvarez G. S. Hélary C. Mebert A. M. Wang X. Coradin T. Desimone M. F. (2014). Antibiotic-loaded silica nanoparticle–collagen composite hydrogels with prolonged antimicrobial activity for wound infection prevention. *J. Mater. Chem. B* 2 4660–4670. 10.1039/c4tb00327f 32262278

[B2] Ananthakrishnan A. N. (2011). *Clostridium difficile* infection: epidemiology, risk factors and management. *Nat. Rev. Gastroenterol. Hepatol.* 8 17–26. 10.1038/nrgastro.2010.190 21119612

[B3] Babakhani F. Bouillaut L. Gomez A. Sears P. Nguyen L. Sonenshein A. L. (2012). Fidaxomicin inhibits spore production in *Clostridium difficile*. *Clin. Infect. Dis.* 55(Suppl. 2), S162–S169. 10.1093/cid/cis453 22752866 PMC3388029

[B4] Baines S. D. O’Connor R. Saxton K. Freeman J. Wilcox M. H. (2009). Activity of vancomycin against epidemic *Clostridium difficile* strains in a human gut model. *J. Antimicrob. Chemother.* 63 520–525. 10.1093/jac/dkn502 19112083

[B5] Bouwknegt M. van Dorp S. Kuijper E. (2015). Burden of *Clostridium difficile* infection in the United States. *N. Engl. J. Med.* 372 2368–2370. 10.1056/NEJMc1505190 26061851

[B6] Deakin L. J. Clare S. Fagan R. P. Dawson L. F. Pickard D. J. West M. R. (2012). The *Clostridium difficile* Spo0A gene is a persistence and transmission factor. *Infect. Immun.* 80 2704–2711. 10.1128/IAI.00147-12 22615253 PMC3434595

[B7] Erben U. Loddenkemper C. Doerfel K. Spieckermann S. Haller D. Heimesaat M. M. (2014). A guide to histomorphological evaluation of intestinal inflammation in mouse models. *Int. J. Clin. Exp. Pathol.* 7 4557–4576. 25197329 PMC4152019

[B8] Francis M. B. Allen C. A. Shrestha R. Sorg J. A. (2013). Bile acid recognition by the *Clostridium difficile* germinant receptor, CspC, is important for establishing infection. *PLoS Pathog.* 9:e1003356. 10.1371/journal.ppat.1003356 23675301 PMC3649964

[B9] Gerding D. N. Johnson S. Rupnik M. Aktories K. (2014). *Clostridium difficile* binary toxin CDT: mechanism, epidemiology, and potential clinical importance. *Gut Microbes* 5 15–27. 10.4161/gmic.26854 24253566 PMC4049931

[B10] Gómez S. Chaves F. Orellana M. A. (2017). Clinical, epidemiological and microbiological characteristics of relapse and re-infection in *Clostridium difficile* infection. *Anaerobe* 48 147–151. 10.1016/j.anaerobe.2017.08.012 28830842

[B11] Gopinath P. M. Dhanasekaran D. Ranjani A. Thajuddin N. Akbarsha M. A. Velmurugan M. (2015). Optimization of sporicidal activity and environmental *Bacillus* endospores decontamination by biogenic silver nanoparticle. *Future Microbiol.* 10 725–741. 10.2217/fmb.14.150 26000648

[B12] Gu H. Ho P. L. Tong E. Wang L. Xu B. (2003). Presenting vancomycin on nanoparticles to enhance antimicrobial activities. *Nano Lett.* 3 1261–1263. 10.1021/nl034396z

[B13] Hassan M. M. Ranzoni A. Phetsang W. Blaskovich M. A. T. Cooper M. A. (2017). Surface ligand density of antibiotic-nanoparticle conjugates enhances target avidity and membrane permeabilization of vancomycin-resistant bacteria. *Bioconjug. Chem.* 28 353–361. 10.1021/acs.bioconjchem.6b00494 27959504

[B14] Hopkins R. J. Wilson R. B. (2018). Treatment of recurrent colitis: a narrative review. *Gastroenterol. Rep.* 6 21–28. 10.1093/gastro/gox041 29479439 PMC5806400

[B15] Huang C.-C. Chuang K.-Y. Chou C.-P. Wu M.-T. Sheu H.-S. Shieh D.-B. (2011). Size-control synthesis of structure deficient truncated octahedral Fe3-δO4 nanoparticles: high magnetization magnetites as effective hepatic contrast agents. *J. Mater. Chem.* 21 7472–7479. 10.1039/c1jm10325c

[B16] Hung Y.-P. Ko W.-C. Chou P.-H. Chen Y.-H. Lin H.-J. Liu Y.-H. (2015). Proton-pump inhibitor exposure aggravates *Clostridium difficile*-associated colitis: evidence from a mouse model. *J. Infect. Dis.* 212 654–663. 10.1093/infdis/jiv184 25805751

[B17] Hussain S. Joo J. Kang J. Kim B. Braun G. B. She Z.-G. (2018). Antibiotic-loaded nanoparticles targeted to the site of infection enhance antibacterial efficacy. *Nat. Biomed. Eng.* 2 95–103. 10.1038/s41551-017-0187-5 29955439 PMC6015743

[B18] Johnson S. Samore M. H. Farrow K. A. Killgore G. E. Tenover F. C. Lyras D. (1999). Epidemics of diarrhea caused by a clindamycin-resistant strain of *Clostridium difficile* in four hospitals. *N. Engl. J. Med.* 341 1645–1651. 10.1056/NEJM199911253412203 10572152

[B19] Joshi L. T. Phillips D. S. Williams C. F. Alyousef A. Baillie L. (2012). Contribution of spores to the ability of *Clostridium difficile* to adhere to surfaces. *Appl. Environ. Microbiol.* 78 7671–7679. 10.1128/AEM.01862-12 22923404 PMC3485709

[B20] Kalghatgi S. Spina C. S. Costello J. C. Liesa M. Morones-Ramirez J. R. Slomovic S. (2013). Bactericidal antibiotics induce mitochondrial dysfunction and oxidative damage in Mammalian cells. *Sci. Transl. Med.* 5:192ra85. 10.1126/scitranslmed.3006055 23825301 PMC3760005

[B21] Kavruk M. Celikbicak O. Ozalp V. C. Borsa B. A. Hernandez F. J. Bayramoglu G. (2015). Antibiotic loaded nanocapsules functionalized with aptamer gates for targeted destruction of pathogens. *Chem. Commun.* 51 8492–8495. 10.1039/c5cc01869b 25891472

[B22] Kell A. J. Stewart G. Ryan S. Peytavi R. Boissinot M. Huletsky A. (2008). Vancomycin-modified nanoparticles for efficient targeting and preconcentration of Gram-positive and Gram-negative bacteria. *ACS Nano* 2 1777–1788. 10.1021/nn700183g 19206416

[B23] Knowles B. R. Yang D. Wagner P. Maclaughlin S. Higgins M. J. Molino P. J. (2018). Zwitterion functionalized silica nanoparticle coatings: the effect of particle size on protein, bacteria, and fungal spore adhesion. *Langmuir* 35 1335–1345. 10.1021/acs.langmuir.8b01550 30086644

[B24] Kwon E. J. Skalak M. Bertucci A. Braun G. Ricci F. Ruoslahti E. (2017). Porous silicon nanoparticle delivery of tandem peptide anti-infectives for the treatment of *Pseudomonas aeruginosa* lung infections. *Adv. Mater.* 29. 10.1002/adma.201701527 28699173 PMC5765747

[B25] Lawley T. D. Clare S. Deakin L. J. Goulding D. Yen J. L. Raisen C. (2010). Use of purified *Clostridium difficile* spores to facilitate evaluation of health care disinfection regimens. *Appl. Environ. Microbiol.* 76 6895–6900. 10.1128/AEM.00718-10 20802075 PMC2953018

[B26] Lee W.-T. Wu Y.-N. Chen Y.-H. Wu S.-R. Shih T.-M. Li T.-J. (2017). Octahedron iron oxide nanocrystals prohibited *Clostridium difficile* spore germination and attenuated local and systemic inflammation. *Sci. Rep.* 7:8124. 10.1038/s41598-017-08387-y 28811642 PMC5558001

[B27] Leffler D. A. Lamont J. T. (2015). *Clostridium difficile* infection. *N. Engl. J. Med.* 372 1539–1548. 10.1056/NEJMra1403772 25875259

[B28] Mora-Uribe P. Miranda-Cárdenas C. Castro-Córdova P. Gil F. Calderón I. Fuentes J. A. (2016). Characterization of the adherence of spores: the integrity of the outermost layer affects adherence properties of spores of the epidemic strain R20291 to components of the intestinal mucosa. *Front. Cell. Infect. Microbiol.* 6:99. 10.3389/fcimb.2016.00099 27713865 PMC5031699

[B29] Morgan M. J. Kim Y.-S. Liu Z. (2008). TNFalpha and reactive oxygen species in necrotic cell death. *Cell Res.* 18 343–349. 10.1038/cr.2008.31 18301379

[B30] Paredes-Sabja D. Sarker M. R. (2012). Adherence of *Clostridium difficile* spores to Caco-2 cells in culture. *J. Med. Microbiol.* 61 1208–1218. 10.1099/jmm.0.043687-0 22595914

[B31] Qi G. Li L. Yu F. Wang H. (2013). Vancomycin-modified mesoporous silica nanoparticles for selective recognition and killing of pathogenic gram-positive bacteria over macrophage-like cells. *ACS Appl. Mater. Interfaces* 5 10874–10881. 10.1021/am403940d 24131516

[B32] Rose H. L. Dewey C. A. Ely M. S. Willoughby S. L. Parsons T. M. Cox V. (2011). Comparison of eight methods for the extraction of *Bacillus atrophaeus* spore DNA from eleven common interferents and a common swab. *PLoS One* 6:e22668. 10.1371/journal.pone.0022668 21818364 PMC3144239

[B33] Sekirov I. Tam N. M. Jogova M. Robertson M. L. Li Y. Lupp C. (2008). Antibiotic-induced perturbations of the intestinal microbiota alter host susceptibility to enteric infection. *Infect. Immun.* 76 4726–4736. 10.1128/IAI.00319-08 18678663 PMC2546810

[B34] Simón-Gracia L. Hunt H. Scodeller P. Gaitzsch J. Kotamraju V. R. Sugahara K. N. (2016). iRGD peptide conjugation potentiates intraperitoneal tumor delivery of paclitaxel with polymersomes. *Biomaterials* 104 247–257. 10.1016/j.biomaterials.2016.07.023 27472162 PMC5687559

[B35] Sorg J. A. Sonenshein A. L. (2010). Inhibiting the initiation of *Clostridium difficile* spore germination using analogs of chenodeoxycholic acid, a bile acid. *J. Bacteriol.* 192 4983–4990. 10.1128/JB.00610-10 20675492 PMC2944524

[B36] Sugahara K. N. Teesalu T. Karmali P. P. Kotamraju V. R. Agemy L. Greenwald D. R. (2010). Coadministration of a tumor-penetrating peptide enhances the efficacy of cancer drugs. *Science* 328 1031–1035. 10.1126/science.1183057 20378772 PMC2881692

[B37] Wagner G. Korenkov V. Judy J. D. Bertsch P. M. (2016). Nanoparticles composed of Zn and ZnO inhibit spore germination and infectivity on tobacco leaves. *Nanomater* 6:E50. 10.3390/nano6030050 28344307 PMC5302510

[B38] Warren C. A. van Opstal E. J. Riggins M. S. Li Y. Moore J. H. Kolling G. L. (2013). Vancomycin treatment’s association with delayed intestinal tissue injury, clostridial overgrowth, and recurrence of *Clostridium difficile* infection in mice. *Antimicrob. Agents Chemother.* 57 689–696. 10.1128/aac.00877-12 23147742 PMC3553708

[B39] Zarkan A. Macklyne H.-R. Truman A. W. Hesketh A. R. Hong H.-J. (2016). The frontline antibiotic vancomycin induces a zinc starvation response in bacteria by binding to Zn(II). *Sci. Rep.* 6:19602. 10.1038/srep19602 26797186 PMC4726154

