# Supplementary material for: Vancomycin-Loaded Nanoparticles Enhance Sporicidal and Antibacterial Efficacy for Clostridium difficile Infection
Source: Front Microbiol. 2019 May 24;10:1141. doi: 10.3389/fmicb.2019.01141 (PMC6543869; doi:10.3389/fmicb.2019.01141)
Supplement: Supplementary file 1 [file Data_Sheet_1.pdf]

Supplementary Materials

1. Supplementary Figures

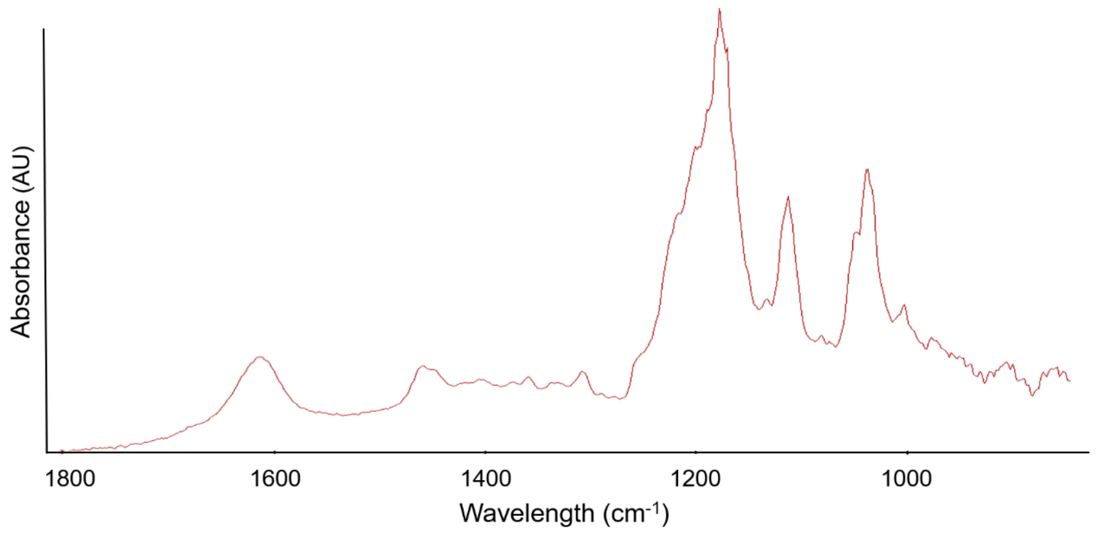

Supplementary Figure 1. The Fourier transform infrared spectroscopy (FTIR) spectra of the van-IONP. The FTIR spectra of van-IONP showed the characteristic peaks of functional group of vancomycin. The peak at 1615 cm^{-1} corresponds to $R–CO–NH_2$. And the peaks in the range between 1350-900 cm^{-1} correspond to R–O–R. The peaks suggest the presence of vancomycin on van-IONP.

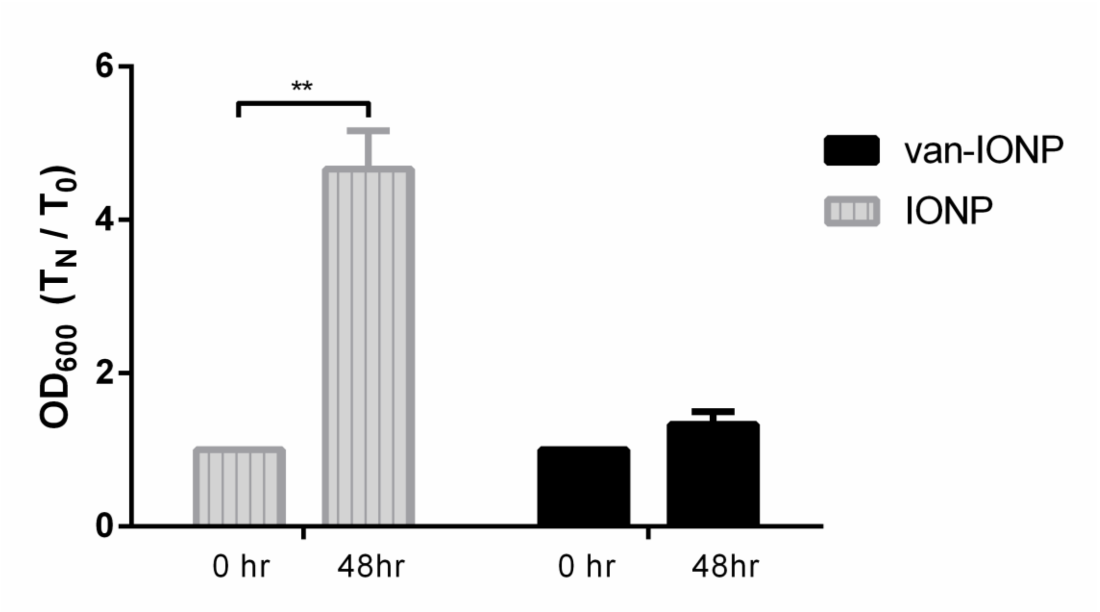

Supplementary Figure 2. The result showed the van-IONP have superior vegetative inhibitory effect than IONP after 48 h incubation, which indicate the bioavailability of vancomycin on van-IONP (n=4). $^{**}P < 0.01$

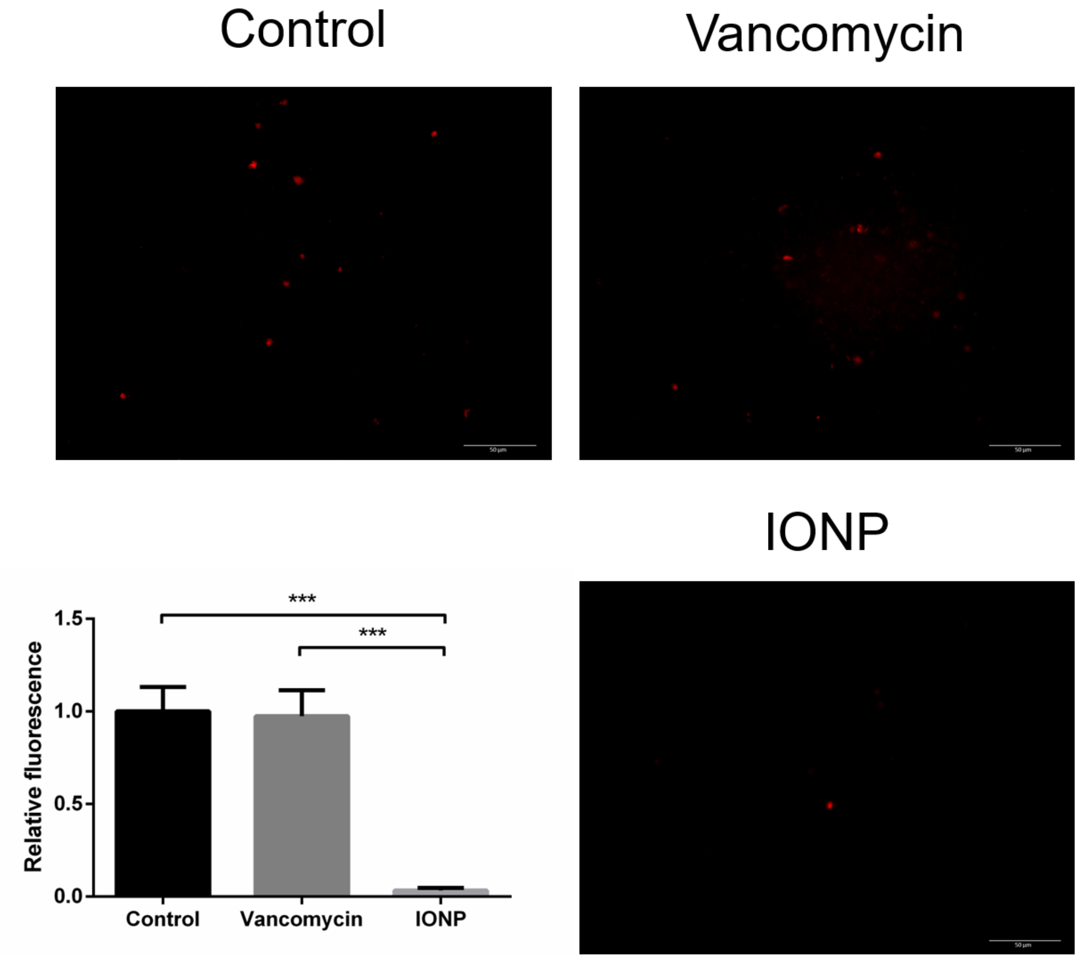

Supplementary Figure 3. Fluorescence micrographs of adhered fluorescent-labeled *C. difficile* spores (red) to monolayers of HT29 cells. The amount adhered to spores was higher in control and vancomycin group than the IONP-treated group. The fluorescence intensity was counted using ImageJ software; the intensity was significantly higher in the control and vancomycin group than in the IONP-treated group (n = 5). $^{***}P < 0.001$

2. Supplementary material and methods

2-1. Vegetative cell outgrowth inhibitory assay

To further test the bioavailability of vancomycin on van-IONP to damage vegetative cells, we perform OD based growth assay according to (Allen et al., 2013). Briefly, 0.1 mL OD600 0.2 vegetative cells of C. difficile BAA-1805 was mixed with van-IONP and IONP to concentration of 3.6 μg/mL respectively in a 96-well plate. After 48 h

incubation in an anaerobic chamber, the OD600 was measured again to measure the growth of vegetative cells. (n = 4)

2.2 Statistics

All data were expressed as the mean ± standard deviations and statistical comparisons among the groups were analyzed by Student's t-test. Multiple intergroup comparisons were assessed by one-way ANOVA, followed by post hoc Tukey's test with GraphPad Prism version 6.0. Statistical significance was set at $P < 0.05$.

3. Reference

Allen, C. A., Babakhani, F., Sears, P., Nguyen, L., and Sorg, J. A. (2013). Both fidaxomicin and vancomycin inhibit outgrowth of Clostridium difficile spores. Antimicrob. agents Chemother. 57, 664–667. doi:10.1128/AAC.01611-12.
